# Serum from pregnant donors induces human beta cell proliferation

**DOI:** 10.1080/19382014.2024.2334044

**Published:** 2024-03-27

**Authors:** Kendra R. Sylvester-Armstrong, Callie F. Reeder, Andrece Powell, Matthew W. Becker, D. Walker Hagan, Jing Chen, Clayton E. Mathews, Clive H. Wasserfall, Mark A. Atkinson, Robert Egerman, Edward A. Phelps

**Affiliations:** a Department of Obstetrics & Gynecology, College of Medicine, University of Florida, Gainesville, Florida, USA; b J. Crayton Pruitt Family Department of Biomedical Engineering, University of Florida, Gainesville, Florida, USA; c Department of Pathology, Immunology, and Laboratory Medicine and University of Florida Diabetes Institute, University of Florida, Gainesville, Florida, USA

**Keywords:** Beta cell, diabetes, EdU, EndoC-βH1, proliferation, human, insulin, islet, pregnancy, primary, serum

## Abstract

Pancreatic beta cells are among the slowest replicating cells in the human body and have not been observed to increase in number except during the fetal and neonatal period, in cases of obesity, during puberty, as well as during pregnancy. Pregnancy is associated with increased beta cell mass to meet heightened insulin demands. This phenomenon raises the intriguing possibility that factors present in the serum of pregnant individuals may stimulate beta cell proliferation and offer insights into expansion of the beta cell mass for treatment and prevention of diabetes. The primary objective of this study was to test the hypothesis that serum from pregnant donors contains bioactive factors capable of inducing human beta cell proliferation. An immortalized human beta cell line with protracted replication (EndoC-βH1) was cultured in media supplemented with serum from pregnant and non-pregnant female and male donors and assessed for differences in proliferation. This experiment was followed by assessment of proliferation of primary human beta cells. Sera from five out of six pregnant donors induced a significant increase in the proliferation rate of EndoC-βH1 cells. Pooled serum from the cohort of pregnant donors also increased the rate of proliferation in primary human beta cells. This study demonstrates that serum from pregnant donors stimulates human beta cell proliferation. These findings suggest the existence of pregnancy-associated factors that can offer novel avenues for beta cell regeneration and diabetes prevention strategies. Further research is warranted to elucidate the specific factors responsible for this effect.

## GRAPHICAL ABSTRACT

**Figure:**
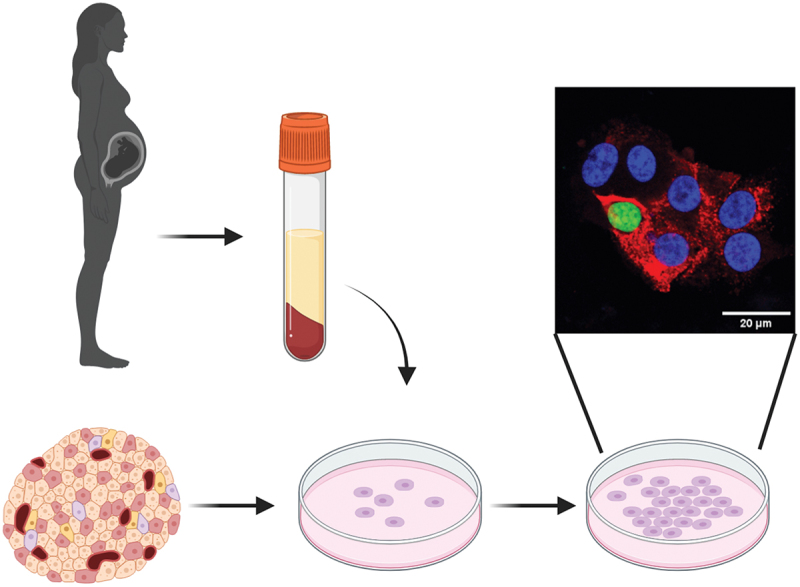

## Introduction

Glucose metabolism changes during pregnancy to support the growing fetus’ needs for glucose through passive transport dependent on a concentration gradient between fetal and maternal circulations. In the later stages of pregnancy, the placenta secretes hormones to increase maternal insulin resistance and hepatic glucose production resulting in elevated circulating maternal glucose to support the gradient.^1^ To prevent excessive nutrient delivery to the fetus and prevent maternal hyperglycemia, pregnancy is also associated with an increase in beta cell mass.^1,2^ Increased beta cell mass during human pregnancy has been investigated by two autopsy series,^3,4^ but has not yet been under intensive investigation in live human adult beta cells. A mechanism for increased beta cell mass during pregnancy (replication, neogenesis, or both) in humans remains uncertain.^1,4,5^

In rodents, pregnancy-associated beta cell expansion depends on secreted placental lactogens that signal through the prolactin receptor.^2^ At the end of pregnancy in rodents, the beta cell population contracts back to its pre-pregnancy size. However, loss of prolactin associated signaling pathways in the mouse islet does not completely block the proliferative response to pregnancy. Studies of lactogens on human beta cell proliferation have generated inconclusive results.^6,7^ Therefore, other signals such as serotonin are likely to contribute to beta cell proliferation in human pregnancy.^5,8–10^

Here, we cultured EndoC-βH1 and primary human islets in the presence of human serum from pregnant donors in the third trimester and within 24–48 h postpartum. We measured proliferation based on 5-ethynyl-2’-deoxyuridine (EdU) incorporation and compared pregnant serum with non-pregnant female serum, fetal bovine serum (FBS), and serum-free defined growth medium.

## Results

### Effect of maternal serum on EndoC-βH1 beta cells

The immortalized human beta cell-line EndoC-βH1 was selected for this study because it is homogenous, transcriptionally and functionally similar to immature primary human beta cells, is cultured in a defined serum-free medium, and maintains a low rate of basal proliferation for up to 100 passages.^11^ We measured the effect of supplementing the cell culture medium with 10% maternal serum on EndoC-βH1 beta cell proliferation by EdU incorporation assay. EdU is a thymidine analogue that is incorporated into DNA during replication and later detected using click chemistry via a copper-catalyzed azide alkyne cycloaddition. The basal proliferation rate and homogeneity of EndoC-βH1 was sufficient for us to implement a plate reader format for the EdU assay, allowing us to measure relative proliferation rates with higher throughput than image-based analysis assays.

**Figure 1.**
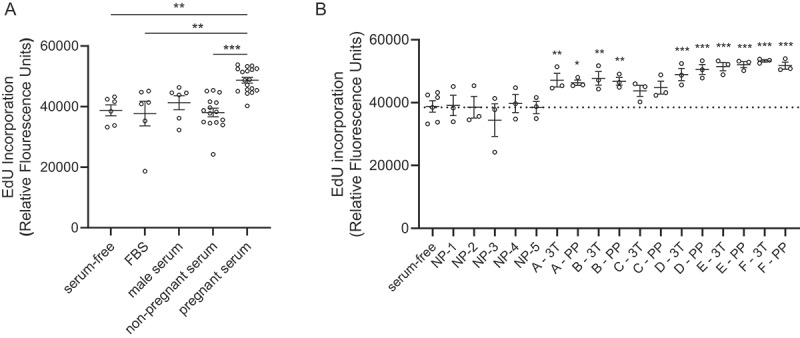
Maternal human serum increases EndoC-βH1 beta cell proliferation. (a) EndoC-βH1 cells were cultured in 96-well plates in defined serum free conditions, supplemented with 10% fetal bovine serum (FBS), 10% male serum, 10% non-pregnant female serum (NP), or 10% pregnant female human serum (3rd trimester) for seven days. Proliferation was measured by fluorescent click-iT EdU proliferation assay for microplates as measured by the fluorescence signal of Amplex UltraRed reagent, an HRP substrate, where HRP is attached to EdU by click chemistry. Data are combined from two experiments run with the same conditions. (b) The EndoC-βH1 proliferation response to pregnant serum collected from 6 individual donors at the third trimester (3T) was compared to 24–48 hours post-partum (PP). Five of six individual donors showed a significant increase in proliferation relative to defined serum-free culture medium. There was no difference in proliferation between 3T and PP for any donor. For both panels, each dot represents the proliferation response in an individual well of a 96 well plate. Panels A and B are the same data, combined by donor type (A) or split out into individual serum donor (B). Groups were assessed for differences by one-way ANOVA with Tukey’s post hoc pairwise comparisons relative to serum-free. Values are mean ± SEM. *p < .05, **p < .01, ***p < .001.

We measured a significant increase in the degree of proliferation for EndoC-βH1 beta cells cultured with 10% maternal serum (third trimester) relative to serum-free medium, 10% FBS, or 10% non-pregnant female serum (Figure 1(a)). Serum donor characteristics are provided in Table 1. Comparing serum collected during the third trimester and 24–48-h post-delivery on a per donor basis, five out of six pregnant donors significantly increased proliferation of EndoC-βH1 beta cells over baseline levels (Figure 1(b)). In contrast, EndoC-βH1 beta cell proliferation was not increased by incubation with serum from individual non-pregnant donors or a pool of serum from male donors (Figure 1(a,b)). Serum collected during the third trimester and within two-days post-partum both imparted similar degrees of beta cell proliferation.

**Table 1.** Characteristics of female^a^ research volunteers.

Donor	Diabetes	Pregnant	Race	Age	GA delivery	BMI delivery	Hyper-tension	GA sample	Glucose	A1c
A − 3T^b^	0	1	White	22	35	30.1	0	34	99.9	5.1
A – PP^c^	0	0	White	22	35	30.1	0	0	79.5	5.4
B − 3T	0	1	White	23	35	20.7	0	34	79.6	5.0
B – PP	0	0	White	23	35	20.7	0	0	93.9	5.3
C − 3T	0	1	White	29	39	32.7	0	39	91.2	5.5
C – PP	0	0	White	29	39	32.7	0	0	82.6	5.5
D − 3T	0	1	White	31	37	28.9	0	37	80.3	5.5
D – PP	0	0	White	31	37	28.9	0	0	80.5	5.6
E − 3T	0	1	White	23	41	46.9	0	41	77.8	5.2
E – PP	0	0	White	23	41	46.9	0	0	73.7	5.1
F − 3T	0	1	African-American	40	36	35.5	1	36	76.5	5.5
F – PP	0	0	African-American	40	36	35.5	1	0	74.3	5.5
NP^d^ − 1	0	0	White	26	n/a	n/a	n/a	n/a	n/a	n/a
NP − 2	0	0	White	31	n/a	22.8	n/a	n/a	92.1	5.1
NP − 3	0	0	African American	30	n/a	22.7	n/a	n/a	57.7	4.7
NP − 4	0	0	White	29	n/a	25.2	n/a	n/a	67.7	5
NP − 5	0	0	African American	24	n/a	20.3	n/a	n/a	82.2	5.2

^a^A pool of serum from males was purchased from a commercial vendor. The data presented in this table were not available for male serum donors.

^b^Third trimester serum collection (3T)

^c^Postpartum 24–48 hours serum collection (PP)

^d^Non-pregnant female (NP)

### Effect of maternal serum on primary human islets

Next, we tested the effect of maternal serum on monolayers of dispersed primary human islets isolated from cadaver donors over a seven day culture. Islet donor characteristics are reported in Table 2. Monolayer culture is a standard technique to accurately identify proliferating primary beta cells,^12–15^ which can be difficult to visualize in intact three-dimensional, whole islets. Dispersion of the islet and culture in monolayer may also encourage proliferation of primary cells by disrupting cell–cell and cell–matrix junctions. Primary islet cultures consist of multiple cell types and beta proliferation events are rare (<1%), where most proliferating cells may be fibroblasts or other non-beta cells. Thus, to improve the cell-type specificity of the EdU proliferation assay, we transitioned to an image-based assay and co-stained for insulin. Insulin positivity alone does not necessarily signify a mature human beta cell. Without staining for additional markers, it is difficult to rule out the possibility of immature beta cells or polyhormonal cells responding to the pregnant serum. Because the availability of primary human islets is limited, we pooled serum from multiple donors rather than attempt to analyze proliferation on a per-donor basis.

**Table 2.** Human islet donor characteristics.

Donor number	Donor RRID	Age	Sex	BMI	HbA1c	Donor type
1	SAMN13028024	57	female	23.5	4.40%	no diabetes
2	SAMN32641505	16	male	29.5	5.40%	no diabetes
3	SAMN38788294	42	male	20.7	5.5%	no diabetes

**Figure 2.**
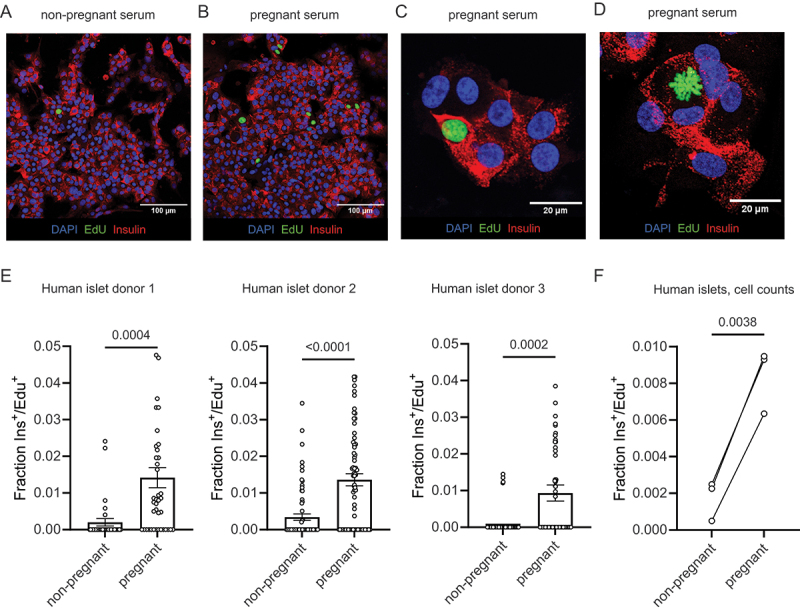
Pregnant human serum increases proliferation in primary human beta cells. (a–c) monolayer preparations of primary human islets were cultured with 10% pregnant or 10% non-pregnant serum from five pooled donors for seven days. Proliferation was detected by imaging of EdU incorporation in insulin positive cells. Panel (c) shows increased magnification verifying presence in insulin^+^/EdU^+^ beta cells. (d) Image shows a mitotic figure with condensed chromosomes in an adult human beta cell treated with pregnant serum verifying that beta cells can proliferate under these conditions, albeit at very low frequency. (e) Image quantification of Ins^+^/EdU^+^ cells. Each dot represents EdU positive beta cells for a single image. Islet donor 1: *N* = 32 and 41 images for non-pregnant and pregnant, respectively, across 9 wells. Islet donor 2: *N* = 71 and 70 images for non-pregnant and pregnant, respectively, across 15 wells. Islet donor 3 *N* = 105 and 112 images for non-pregnant and pregnant, respectively, across 8 wells). Groups were assessed for differences by two-tailed t test, p-values are shown on the plots. (f) Image quantification shown as the total cells counted across all images. Each dot represents the fraction of Ins^+^/EdU^+^ cells from all cells imaged in all wells in one islet donor. Groups were assessed for differences by two-tailed t test, p-value is shown on the plot.

After one week of incubation with pooled serum from pregnant and non-pregnant donors, human islet cell monolayers were fixed, immunostained for insulin, and developed for EdU incorporation (Figure 2(a–d)). As is typical for primary human islets, beta cell proliferation was extremely low across all samples, requiring confocal imaging at 20× or higher magnification to identify proliferating cells. By capturing multiple images per well and quantifying the insulin^+^/Edu^+^ double-positive cells per frame, we observed a significantly higher percentage of proliferating beta cells when cultured with pregnant serum (Figure 2(e,f)). Islet proliferation responses by islet donor are reported in Table 3.

**Table 3.** Human islet monolayer total cell counts following treatment with pooled pregnant or non-pregnant human serum. Data correspond to the plot in figure 2F.

	Islet donor 1		Islet donor 2		Islet donor 3	
	Non-pregnant	Pregnant	Non-pregnant	Pregnant	Non-pregnant	Pregnant
total β cells	3547	4430	11250	10218	8028	7723
EdU^+^ β cells	8	42	28	95	4	49
fraction Ins^+^/EdU^+^	0.0023	0.0095	0.0025	0.0093	0.0005	0.0063

**Figure 3.**
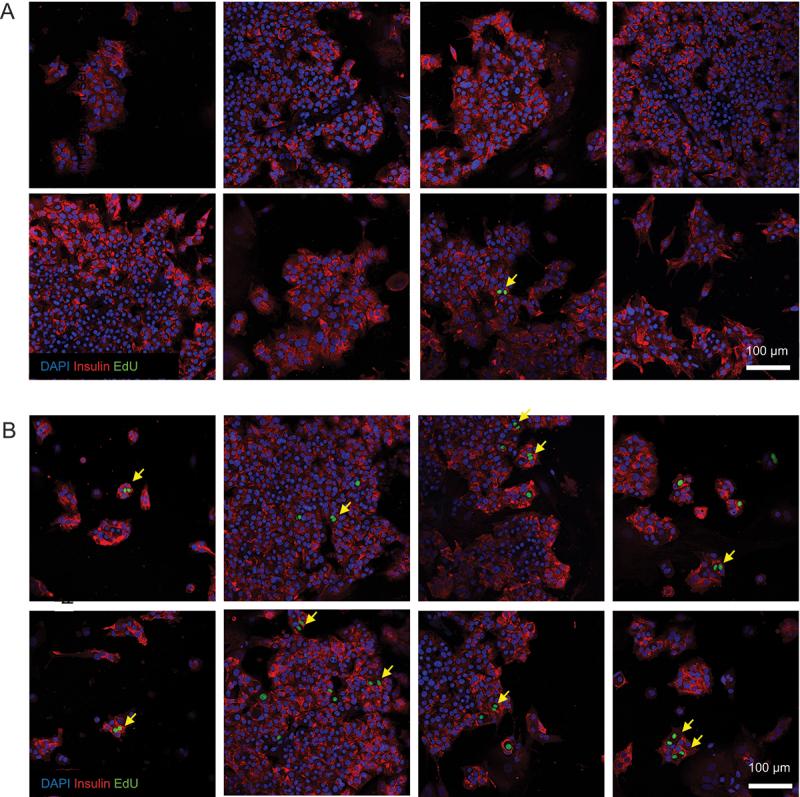
Beta cell nuclear doublets in pregnant serum-treated beta cells. Several representative images of beta cell monolayers stained for insulin, EdU incorporation and DAPI for nuclei following culture with 10% non-pregnant (A) or pregnant (B) human serum from pooled donors for one week. The images in panel (B) show evidence of primary human beta cell proliferation by the observation of EdU^+^ cells frequently occurring as doublets (yellow arrows), indicative of two neighboring daughter cells arising from a recent cell division event.

There is some disagreement as to whether adult human beta cells are capable of cell division. We offer evidence of adult human beta cell replication in our culture system through the observation of rare insulin^+^/EdU^+^ mitotic figures (Figure 2(d)) and insulin^+^/EdU^+^ cell doublets (Figure 3).

## Discussion

This study is the first to our knowledge to investigate the effect of human maternal serum on human beta cell proliferation. An in vitro proliferation response to pregnant human serum has been observed for rat beta cells^16^ but not previously for human beta cells. The peak neonatal replication for rodent beta cells is ~10%–30% while in humans the peak beta cell replication maxes out during the neonatal period at ~2%.^17–21^ This marked difference in species beta cell proliferation rate makes replication of primary human beta cells much more challenging to observe. In our case, we only observed rare proliferative events.

The serum of pregnant donors enhanced the proliferation rate of EndoC-βH1 beta cells by 10–20%. Our selection of the immortalized EndoC-βH1 human beta cell line was intentional due to its intrinsic proliferation rate. However, EndoC-βH1 significantly diverges from the biology of primary human beta cells. The recently improved human beta cell-line EndoC-βH5, which is not immortalized, could be implemented in future work to augment studies in primary human islets.

Here, pooled serum from pregnant donors triggered small increases in proliferation of primary human beta cells from 0.05%–0.25% (non-pregnant serum) to 0.63%–0.95% (pregnant serum). This proliferative response was observed in islets from three unique donors (both sexes, 1 female and 2 male, and across a wide age range, 16–57 years) but should be repeated in more islet donors in future work to understand if the proliferation response is correlated with particular characteristics such as age or sex. While the number of replicated primary human beta cells induced by our study with maternal human serum is low, the increase in proliferation rate is similar to the values reported for harmine, one of the more successful beta cell mitogens.^12^ Harmine is known to also cause proliferation of non-beta cells within the islet.^22^ We did observe occasional insulin^neg^/Edu^+^ cells, which would likely be non-beta endocrine cells in our culture. As harmine treatment can increase beta cell mass in vivo sufficiently to improve glycemic control,^12^ the beta cell proliferation induced by pregnant serum in our study is likely to be biologically significant.

We have not yet determined the identity of the factor or factors in pregnant human serum responsible for the increase in beta cell proliferation. In mice, the pregnancy-related factors growth hormone, prolactin, insulin-like growth factor I, placental lactogen, adiponectin, and serotonin have been shown to increase mouse beta cell proliferation.^9,23–26^ However, human beta cells are more refractory to proliferation than mouse beta cells. Pregnancy-related factors that induce beta cell proliferation in mice have proven more difficult to observe in human beta cells.^17^ This may be due to differences between mouse and human beta cell biology and/or the higher donor age and low proliferation rate of available human islets. Although much is known about mitogens which enhance proliferation of the mouse islet, loss or blocking individual pathways does not completely block the beta cell response to pregnancy in mice.^5,8,9^ Therefore, multiplexing of signals almost certainly contributes to beta cell proliferation in mouse pregnancy. In humans, a complex cascade of hormones and growth factors contributes to a state of increasing insulin resistance to maintain the maternal-fetal glucose gradient.^27^

We hypothesized that the period of peak insulin resistance and insulin output in late gestation would be a good timepoint to investigate beta cell proliferation.^28^ Our study collected serum from the third trimester and 24–48 h post-partum, but not other time points. We did not observe a disappearance of the proliferative response in serum collected 24–48 h postpartum, indicating serum from a later timepoint might be necessary to observe the switch back to normal physiology. In addition, some pregnancy hormones that increase beta cell proliferation in mice increase up to and beyond delivery (e.g. prolactin). It should be noted that serum from one of the pregnant donors did not induce a proliferative effect on EndoC-βH1 cells. Collected information failed to distinguish this donor from the other five we studied. We believe that a variable donor response is to be expected based on intrinsic heterogeneity in the human population. It could also be due to minor differences in individual serum sample draw, processing, or handling prior to storage. It will be important to complete a longitudinal study of beta cell proliferation responses to serum collected throughout pregnancy and the postpartum period to build a more comprehensive understanding of this process.

An additional limitation of this study is that it was focused on *in vitro* analyses which does not capture the full complexity of physiological organ systems, especially during pregnancy. Enhanced insulin output during human pregnancy may also be a result of increased beta cell function in addition to proliferation. Nevertheless, the study focused exclusively on human cells including primary human beta cells and serum from pregnant human donors which provides additional insight into human beta cell adaptation to pregnancy. Our observations suggest that proliferative serum factors present during human pregnancy may have translational potential for human diabetes therapy.

## Materials and methods

### Study subjects and sample collection

Subjects were recruited at the University of Florida Health Shands Hospital High-Risk Maternal-Fetal Medicine Clinic under a protocol approved by the University of Florida Institutional Review Board (UF IRB20150007, IRB201902512) and in compliance with guidelines for human research outlined in the Declaration of Helsinki. All study subjects gave their written, informed consent for participation prior to enrollment and sample collection. Maternal blood was collected at the third trimester and within two-day post-partum. Samples were collected in conjunction with routine clinical blood draws. Blood was collected in red-top vacutainer tubes and allowed to clot for 1 h at room temperature. Specimens were then centrifuged for 10 min at 3000× g at 4°C, and 400 µL serum aliquots were transferred into 1.8 mL cryovials and stored at −80°C. Pooled serum from male donors was purchased from a commercial vendor (Lonza).

### EndoC-βH1 beta cell culture

The EndoC-βH1 human beta cell line was cultured consistent with conditions described by Tsonkova et al.^11^ The EndoC-βH1 line was cultured in DMEM with low glucose (1 g/L), 2% albumin from bovine serum fraction V, 50 µM 2-mercaptoethanol, 10 mM nicotinamide, 5.5 μg/ml transferrin, 6.7 ng/ml sodium selenite and penicillin (100 units/ml)/streptomycin (100 µg/ml) and maintained in sub-confluent densities. EndoC-βH1 cells (passage 54) were seeded at 2 × 10^5^ cells per well in a cell culture treated 96-well plate and allowed to adhere for 24 h prior to starting of the experiments. The next day, seeding media was exchanged for fresh media containing 10% human serum from pregnant or non-pregnant donors together with EdU (Click-iT™ EdU Proliferation Assay for Microplates, ThermoFisher Scientific) and cultured for 1 week. EdU was detected following the kit manufacturer’s protocol and fluorescence was measured with a microplate reader (Molecular Devices SpectraMax M5) at 568/585 nm.

### Primary human beta cells

We generated monolayer cultures of primary human islet cells as previous reported.^15^ Primary human pancreatic islets from deceased nondiabetic donors were obtained from the Integrated Islet Distribution Program (IIDP) at City of Hope. Human islets were cultured at 24°C and 5% CO_2_ in 10 cm non-adherent cell culture dishes in CMRL medium with 2% L-glutamine, 10% fetal bovine serum (FBS), 10 mM HEPES and 1% penicillin/streptomycin. Islets were dissociated into a suspension of single islet cells by continuous gentle pipetting in 0.3 ml 0.05% trypsin-EDTA per 500 islets for 3 min at 37°C. Islet single cells were seeded 35,000 cells per cm^2^ on coverslip bottom 96 well microplates (Ibidi). Wells were precoated with purified human collagen IV (MilliporeSigma) at 50 μg/ml in HBSS with Ca^2+^/Mg^2+^ for 1 h at 37°C. Islet cells required 3–4 d of culture to adhere and spread on surfaces before further experimentation. Islet monolayers were cultured in minimum essential medium (MEM) with GlutaMAX, 11 mM glucose, 5% FBS, 1 mM sodium pyruvate, 10 mM HEPES, and 1× B-27 supplement. Once primary human islet monolayers were established, 10% pooled human serum from pregnant or non-pregnant donor groups was added together with EdU (Click-iT EdU Cell Proliferation kit for Imaging, Thermo Fisher Scientific) and cultured for 1 week. Samples from the primary human monolayer experiment were fixed and immunostained for insulin with guinea pig anti-insulin primary antibody (Dako A0564) and Alexa-Fluor 568 goat anti-guinea pig secondary antibody (Thermo Fisher Scientific), developed for EdU detection following the kit manufacturer’s protocol, and counterstained with DAPI. Samples were imaged on a Leica SP8 Confocal microscope with a 20×/0.8 numerical aperture Plan-Apochromat air objective.

### Statistical analysis

All data are presented as the mean ± SEM. The statistical significance was calculated by analysis of variance (ANOVA) followed by post-hoc pairwise comparisons for groups of three of more and Student’s t test (two tailed) for groups of two or more. Statistical significance was defined as at least *p* < 0.05. The analysis was performed in GraphPad Prism version 9.0.

## Supplementary Material

pregnant serum graphical abstract.png

## Data and resource availability

All data points generated or analyzed during this study are included in the published article (and its online supplementary files). Human serum donor information is listed in Table 1 and islet donor information is listed in Table 2. Further information on the primary human islets analyzed are available in the IIDP repository using the provided RRID numbers: https://iidp.coh.org/[RRID: SAMN13028024; SAMN32641505, SAMN38788294]. No other applicable resources were generated.

## Preprint

The submitted version of this manuscript has been posted on the BioRxiv preprint server under DOI 10.1101/2023.04.17.537214.

## References

[1] Rieck S, Kaestner KH. Expansion of β-cell mass in response to pregnancy. Trends Endocrinol Metab. 2010;21(3):151–10. doi:10.1016/j.tem.2009.11.001. 20015659 PMC3627215

[2] Baeyens L, Hindi S, Sorenson RL, German MS. β-cell adaptation in pregnancy. Diabetes Obes Metab. 2016;18(Suppl S1):63–70. doi:10.1111/dom.12716. 27615133 PMC5384851

[3] Van Assche FA, Aerts L, De Prins F. A morphological study of the endocrine pancreas in human pregnancy. Br J Obstet Gynaecol. 1978;85(11):818–820. doi:10.1111/j.1471-0528.1978.tb15835.x. 363135

[4] Butler AE, Cao-Minh L, Galasso R, Rizza RA, Corradin A, Cobelli C, Butler PC. Adaptive changes in pancreatic beta cell fractional area and beta cell turnover in human pregnancy. Diabetologia. 2010;53(10):2167–2176. doi:10.1007/s00125-010-1809-6. 20523966 PMC2931643

[5] Baeyens L, Lemper M, Staels W, De Groef S, De Leu N, Heremans Y, German MS, Heimberg H. (Re)generating human beta cells: status, pitfalls, and perspectives. Physiol Rev. 2018;98(3):1143–1167. doi:10.1152/physrev.00034.2016. 29717931 PMC6088144

[6] Chen H, Kleinberger JW, Takane KK, Salim F, Fiaschi-Taesch N, Pappas K, Parsons R, Jiang J, Zhang Y, Liu H. et al. Augmented Stat5 signaling bypasses multiple impediments to lactogen-mediated proliferation in human β-cells. Diabetes. 2015;64(11):3784–3797. doi:10.2337/db15-0083. 26159175 PMC4613973

[7] Brelje TC, Scharp DW, Lacy PE, Ogren L, Talamantes F, Robertson M, Friesen HG, Sorenson RL. Effect of homologous placental lactogens, prolactins, and growth hormones on islet B-cell division and insulin secretion in rat, mouse, and human islets: implication for placental lactogen regulation of islet function during pregnancy. Endocrinology. 1993;132(2):879–887. doi:10.1210/endo.132.2.8425500. 8425500

[8] Kim H, Toyofuku Y, Lynn FC, Chak E, Uchida T, Mizukami H, Fujitani Y, Kawamori R, Miyatsuka T, Kosaka Y. et al. Serotonin regulates pancreatic beta cell mass during pregnancy. Nat Med. 2010;16(7):804–808. doi:10.1038/nm.2173. 20581837 PMC2921604

[9] Moon JH, Kim H, Park J, Choi W, Hong HJ, Ro HJ, Jun S, Choi SH, Banerjee RR, Shong M. et al. Lactation improves pancreatic β cell mass and function through serotonin production. Sci Transl Med. 2020;12(541). doi:10.1126/scitranslmed.aay0455. PMC8276761 32350130

[10] Moon JH, Kim YG, Kim K, Osonoi S, Wang S, Saunders DC, Wang J, Yang K, Kim H, Lee J. et al. Serotonin regulates adult β-cell mass by stimulating perinatal β-cell proliferation. Diabetes. 2020;69(2):205–214. doi:10.2337/db19-0546. 31806625 PMC6971487

[11] Tsonkova VG, Sand FW, Wolf XA, Grunnet LG, Kirstine Ringgaard A, Ingvorsen C, Winkel L, Kalisz M, Dalgaard K, Bruun C. et al. The EndoC-βH1 cell line is a valid model of human beta cells and applicable for screenings to identify novel drug target candidates. Mol Metab. 2018;8:144–157. doi:10.1016/j.molmet.2017.12.007. 29307512 PMC5985049

[12] Wang P, Alvarez-Perez J-C, Felsenfeld DP, Liu H, Sivendran S, Bender A, Kumar A, Sanchez R, Scott DK, Garcia-Ocaña A. et al. A high-throughput chemical screen reveals that harmine-mediated inhibition of DYRK1A increases human pancreatic beta cell replication. Nat Med. 2015;21(4):383–388. doi:10.1038/nm.3820. 25751815 PMC4690535

[13] Aamodt KI, Aramandla R, Brown JJ, Fiaschi-Taesch N, Wang P, Stewart AF, Brissova M, Powers AC. Development of a reliable automated screening system to identify small molecules and biologics that promote human β-cell regeneration. Am J Physiol-Endoc M. 2016;311(5):E859–E868. doi:10.1152/ajpendo.00515.2015. PMC5130356 27624103

[14] Walpita D, Hasaka T, Spoonamore J, Vetere A, Takane KK, Fomina-Yadlin D, Fiaschi-Taesch N, Shamji A, Clemons PA, Stewart AF. et al. A human islet cell culture system for high-throughput screening. SLAS Discovery. 2012;17(4):509–518. doi:10.1177/1087057111430253. PMC5546101 22156222

[15] Phelps EA, Cianciaruso C, Santo-Domingo J, Pasquier M, Galliverti G, Piemonti L, Berishvili E, Burri O, Wiederkehr A, Hubbell JA. et al. Advances in pancreatic islet monolayer culture on glass surfaces enable super-resolution microscopy and insights into beta cell ciliogenesis and proliferation. Sci Rep. 2017;7(1). doi:10.1038/srep45961. PMC5388888 28401888

[16] Nalla A, Ringholm L, Sørensen SN, Damm P, Mathiesen ER, Nielsen JH. Possible mechanisms involved in improved beta cell function in pregnant women with type 1 diabetes. Heliyon. 2020;6(8):e04569. doi:10.1016/j.heliyon.2020.e04569. 32904239 PMC7452446

[17] Wang P, Fiaschi-Taesch NM, Vasavada RC, Scott DK, García-Ocaña A, Stewart AF. Diabetes mellitus—advances and challenges in human β-cell proliferation. Nat Rev Endocrinol. 2015;11(4):201–212. doi:10.1038/nrendo.2015.9. 25687999

[18] Kassem SA, Ariel I, Thornton PS, Scheimberg I, Glaser B. Beta-cell proliferation and apoptosis in the developing normal human pancreas and in hyperinsulinism of infancy. Diabetes. 2000;49(8):1325–1333. doi:10.2337/diabetes.49.8.1325. 10923633

[19] Meier JJ, Butler AE, Saisho Y, Monchamp T, Galasso R, Bhushan A, Rizza RA, Butler PC. β-cell replication is the primary mechanism subserving the postnatal expansion of β-cell mass in humans. Diabetes. 2008;57(6):1584–1594. doi:10.2337/db07-1369. 18334605 PMC3697779

[20] Kohler CU, Olewinski M, Tannapfel A, Schmidt WE, Fritsch H, Meier JJ. Cell cycle control of β-cell replication in the prenatal and postnatal human pancreas. Am J Physiol Endocrinol Metab. 2011;300(1):E221–230. doi:10.1152/ajpendo.00496.2010. 20978233

[21] Gregg BE, Moore PC, Demozay D, Hall BA, Li M, Husain A, Wright AJ, Atkinson MA, Rhodes CJ. Formation of a human β-cell population within pancreatic islets is set early in life. J Clin Endocr Metab. 2012;97(9):3197–3206. doi:10.1210/jc.2012-1206. 22745242 PMC3431572

[22] Maachi H, Ghislain J, Tremblay C, Poitout V. Pronounced proliferation of non-beta cells in response to beta-cell mitogens in isolated human islets of Langerhans. Sci Rep. 2021;11(1):11283. doi:10.1038/s41598-021-90643-3. 34050242 PMC8163757

[23] Bernal-Mizrachi E, Kulkarni RN, Scott DK, Mauvais-Jarvis F, Stewart AF, Garcia-Ocaña A. Human β-cell proliferation and intracellular signaling part 2: still driving in the dark without a road map. Diabetes. 2014;63(3):819–831. doi:10.2337/db13-1146. 24556859 PMC3931400

[24] Bouwens L, Rooman I. Regulation of pancreatic beta-cell mass. Physiol Rev. 2005;85(4):1255–1270. doi:10.1152/physrev.00025.2004. 16183912

[25] Retnakaran R. Adiponectin and β-cell adaptation in pregnancy. Diabetes. 2017;66(5):1121–1122. doi:10.2337/dbi17-0001. 28507212

[26] Qiao L, Wattez J-S, Lee S, Nguyen A, Schaack J, Hay WW, Shao J. Adiponectin deficiency impairs maternal metabolic adaptation to pregnancy in mice. Diabetes. 2017;66(5):1126–1135. doi:10.2337/db16-1096. 28073830 PMC5399613

[27] Kampmann U, Knorr S, Fuglsang J, Ovesen P. Determinants of maternal insulin resistance during pregnancy: an updated overview. J Diabetes Res. 2019;2019:5320156. doi:10.1155/2019/5320156. 31828161 PMC6885766

[28] Catalano PM, Drago NM, Amini SB. Longitudinal changes in pancreatic β-cell function and metabolic clearance rate of insulin in pregnant women with normal and abnormal glucose tolerance. Diabetes Care. 1998;21(3):403–408. doi:10.2337/diacare.21.3.403. 9540023

