# Supplementary figures and images for: Serum from pregnant donors induces human beta cell proliferation
Source: Islets. 2024 Mar 27;16(1):2334044. doi: 10.1080/19382014.2024.2334044 (PMC10978022; doi:10.1080/19382014.2024.2334044)

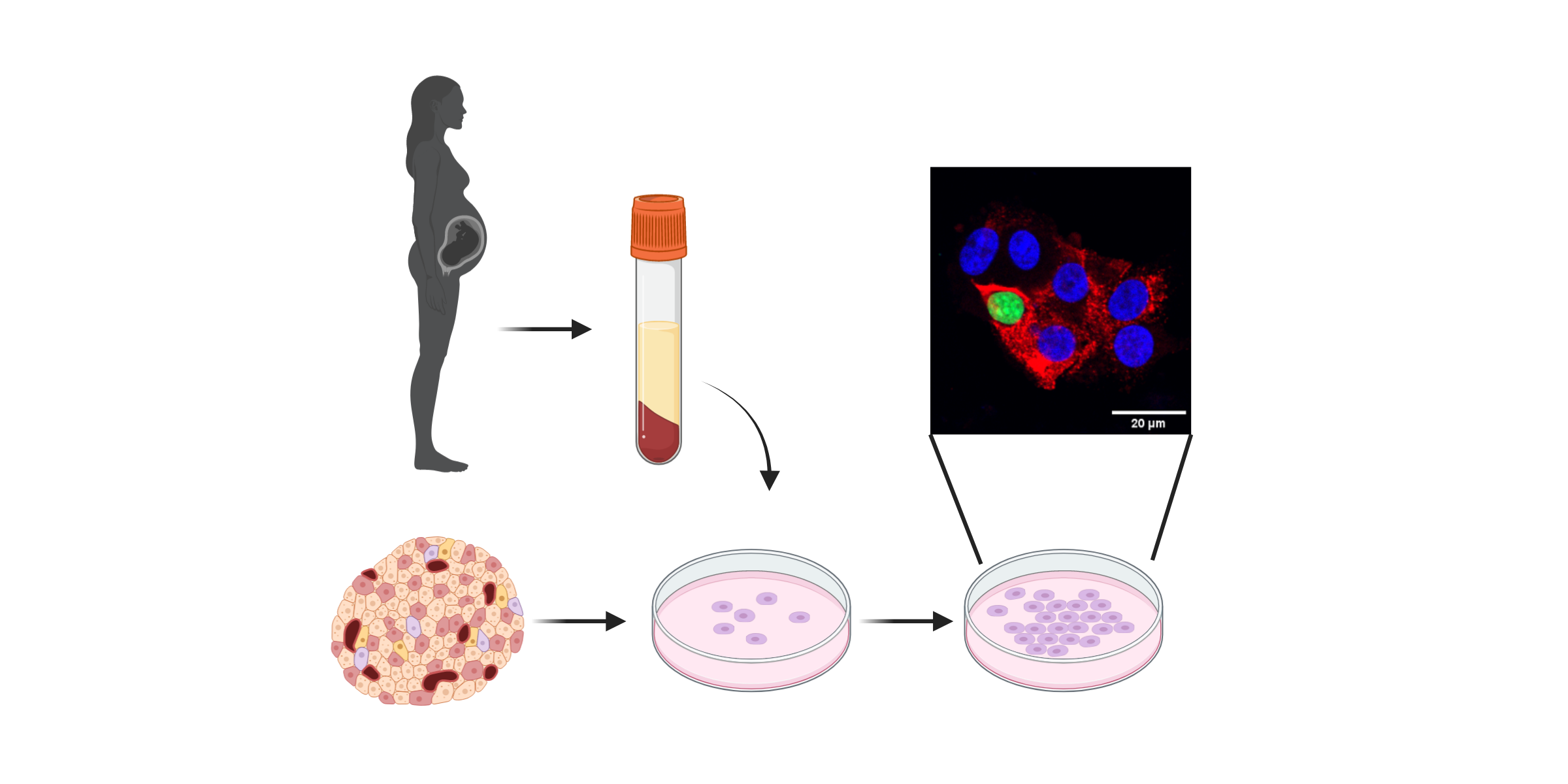

Supplement: pregnant serum graphical abstract.png [file KISL_A_2334044_SM3522.png]
